# Intraoperative Fluid Balance and Perioperative Complications in Ovarian Cancer Surgery

**DOI:** 10.1245/s10434-024-16246-0

**Published:** 2024-10-08

**Authors:** Eva K. Egger, Janina Ullmann, Tobias Hilbert, Damian J. Ralser, Laura Tascon Padron, Milka Marinova, Matthias Stope, Alexander Mustea

**Affiliations:** 1 Department of Gynecology and Gynecological Oncology, University Hospital, Bonn, Germany; 2 Department of Anesthesiology and Intensive Care Medicine, University Hospital Bonn, Bonn, Germany; 3 Department of Nuclear Medicine, University Hospital, Bonn, Germany

**Keywords:** Ovarian cancer, Fluid overload, Fluid restriction, Anastomotic leakage, Complications

## Abstract

**Background:**

Fluid overload and hypovolemia promote postoperative complications in patients undergoing cytoreductive surgery for ovarian cancer. In the present study, postoperative complications and anastomotic leakage were investigated before and after implementation of pulse pressure variation-guided fluid management (PPVGFM) during ovarian cancer surgery.

**Patients and Methods:**

A total of *n *= 243 patients with ovarian cancer undergoing cytoreductive surgery at the University Hospital Bonn were retrospectively evaluated. Cohort A (CA; *n *= 185 patients) was treated before and cohort B (CB; *n *= 58 patients) after implementation of PPVGFM. Both cohorts were compared regarding postoperative complications.

**Results:**

Ultrasevere complications (G4/G5) were exclusively present in CA (*p *= 0.0025). No difference between cohorts was observed regarding severe complications (G3–G5) (*p *= 0.062). Median positive fluid excess was lower in CB (*p *= 0.001). This was independent of tumor load [peritoneal cancer index] (*p *= 0.001) and FIGO stage (*p *= 0.001). Time to first postoperative defecation was shorter in CB (CB: d2 median versus CA: d3 median; *p *= 0.001). CB had a shorter length of hospital stay (*p *= 0.003), less requirement of intensive medical care (*p *= 0.001) and postoperative ventilation (*p *= 0.001). CB received higher doses of noradrenalin (*p *= 0.001). In the combined study cohort, there were more severe complications (G3–G5) in the case of a PFE ≥ 3000 ml (*p *= 0.034) and significantly more anastomotic leakage in the case of a PFE ≥ 4000 ml (*p *= 0.006).

**Conclusions:**

Intraoperative fluid reduction in ovarian cancer surgery according to a PPVGFM is safe and significantly reduces ultrasevere postoperative complications. PFEs of ≥ 3000 ml and ≥ 4000 ml were identified as cutoffs for significantly more severe complications and anastomotic leakage, respectively.

Epithelial ovarian cancer is the most lethal gynecologic malignancy.^1^ Despite recent advantages in systemic therapy, optimal cytoreductive surgery with no postoperative residual tumor represents the most important prognostic parameter.^2–4^ Depending on the surgical complexity needed to reach this goal, the rate of postoperative complications and anastomotic leakage increases steadily.^5,6^

Patients’ age and comorbidities are fixed preoperative risk factors for postoperative complications.^7,8^ Intraoperative fluid management is a modifiable risk factor regarding postoperative complications. Research has identified fluid overload and hypovolemia to promote postoperative complications.^6,9,10^ In colorectal surgery, the Enhanced Recovery After Surgery Society (ERAS) guidelines recommend to aim for a near to zero fluid balance and thereby avoiding anastomotic leakage and further postoperative complications.^11^ Hemodynamic management guidelines for ovarian cancer surgery comparable with the ERAS guidelines are sparse. The European Society of Gynecologic Oncology (ESGO) only recommends a “balanced crystalloid replacement” in their perioperative management guidelines for ovarian cancer surgery.^12^

Extensive peritoneal tumor spread, ascites, hypoproteinemia, and increased expression of vascular leakage genes lead to severe intraoperative volume shifts from the intravascular to the intraabdominal and interstitial space.^13^ Therefore, an optimal hemodynamic intraoperative management avoiding hypovolemia or fluid overload remains challenging.^6,14,15^

The present study compares postoperative outcomes of patients undergoing cytoreductive surgery for ovarian cancer before and after implementation of pulse pressure variation-guided fluid management (PPVGFM).

## Patients and Methods

### Data Collection

This observational retrospective study was conducted in accordance with the standards of the ethics committee of the Faculty of Medicine at the University of Bonn, Germany (no.: 14/22). A total of *n *= 243 patients were retrospectively identified undergoing cytoreductive surgery for ovarian cancer between January 2015 and December 2022 at the University Hospital Bonn. Informed consent for the use of the data was obtained within the framework of our biobank initiative.

### Patients and Variables

Patients’ charts, anesthesiologic protocols, surgery reports, and pathologic findings were screened for general patient characteristics including the preoperative morbidity expressed by the American Society of Anesthesiologists (ASA) score, time of surgery (upfront or interval surgery after neoadjuvant chemotherapy/surgery for primary disease or recurrence), surgical complexity score, tumor load expressed by peritoneal cancer index (PCI) and by the International Federation of Gynecology and Obstetrics (FIGO) stage, performance of anastomoses, intraoperative anesthesiologic and fluid management (noradrenalin use, amount of used crystalloids, colloids, and blood products), and pre- and postoperative hemoglobin and hematocrit levels.

### Treatments

According to the implementation of a pulse pressure variation-guided hemodynamic management (PPVGHM) in 01/2021, patients were separated into cohort A (CA), treated between 01/2015 and 12/2020, and cohort B (CB), treated after implementation of PPVGHM between 01/2021 and 12/2022.

**Fig. 1:**
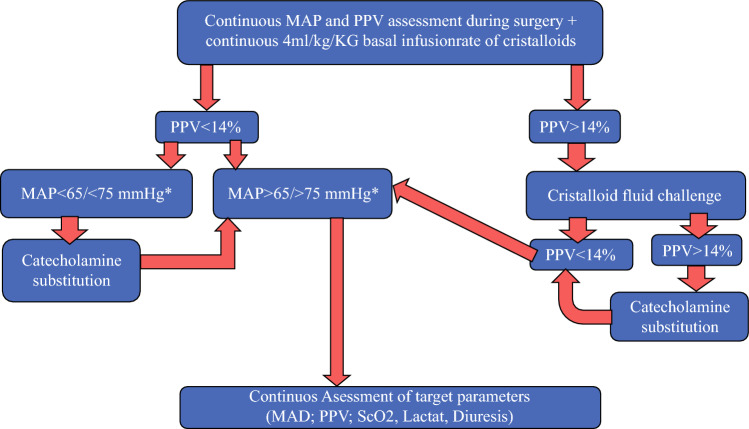
Flow diagram of the PPV-guided hemodynamic intraoperative management * In case of a preoperative pressure above 150 mmHg *MAP* mean arterial pressure, *PPV* positive pulse variation, *ScO*_*2*_ central venous oxygenation

The PPVGHM is shown in Fig. 1. In detail, CB received a basal infusion rate of crystalloids at 4 ml/kg bodyweight. Mean arterial pressure (MAP) was maintained above 65 mmHg or rather above 75 mmHg in case of systolic pressures of 150 mmHg preoperatively and regulated by intravenous noradrenalin dosing, starting at 1 µg/ml/min. Pulse pressure variations (PPV) above 14% were challenged with a crystalloid volume bolus of 250 ml (Jonosteril ®). Missing PPV declines led to intravenous noradrenalin dosing instead of fluid replacement. Further target parameters were central venous oxygenation (ScvO_2_) > 65 %, lactate < 2.0 mmol/l, and diuresis > 0.5 ml/h kg. Blood loss was substituted early by colloids (Gelafundin 4%®) and transfusion of blood products as fresh frozen plasma (FFP) and whole blood transfusions on an individual decision-making process. In the case of 5–l volume substitution, an extended coagulation analysis and albumin analysis was performed. Extubation was always attempted. Furthermore, all patients received a preoperative antibiotic bowel preparation with 400 mg metronidazole three times per day on the day before surgery as well as 45 ml phosphosoda once.

The following anesthesiologic aspects applied to all treated patients:

the placement of a thoracal peridural catheter whenever possible (loaded with 10 ml Naropin 0.375% and 10 µg Sufenta epidural 30 min before the start of surgery and continuous intraoperative loading with Naropin 0.2%, 6–10 ml/h).

Anesthesia was dosed according to a bispectral index value of 40–60%, using remifentanil and continuous propofol infusion of 10 mg/ml or sevoflurane (End tidal concentration (Fet) 1.2%).

A five-lead electrocardiogram and pulse oximetry were used. Preoperative antibiotic administration (cefuroxime 1.5 g/metronidazole 500 mg or clindamycin 600 mg/metronidazole 500 mg) was performed immediately before the start of surgery and repeated after every 3–4 h. All patients received an arterial catheter placement (femoral or radial) for invasive blood pressure measurement, one central intravenous catheter placement, and two peripheral intravenous catheter placements. Furthermore, patients received 8 mg dexamethasone intravenously before the start of surgery. In case of ascites a rapid sequence induction was performed. All patients received a nasogastric tube for the time of surgery, which was removed at the end of surgery. Fluid management in CA was individually applied by the assigned anesthesiologist. The following ERAS instruments have been implemented in all patients: whenever possible a peridural catheter was placed. Guided early mobilization was started at the day after surgery. As soon as independent mobilization was safely performed, the urine catheter was removed. Patients without anastomoses were allowed to eat and drink 6 h after the end of surgery. Patients with anastomoses were allowed to drink after 6 h and fluid feeding started on the first day after surgery in the morning. The nasogastric tube was generally removed at the end of surgery. The central venous catheter was aimed to be removed latest by the third postoperative day.

### Outcome Variables

The primary outcome variable regarding safety was severe postoperative complications within the first 30 days after surgery, graded according to the Memorial Sloan Kettering Cancer Center secondary surgical event score (grade 1 to grade 5; G1–G5).^16^ Further outcome variables were the incidence of anastomotic leakage (AL) and the time to first postoperative defecation. AL was defined by an extravasation of water-soluble contrast agent visible on abdominal computed tomography (CT) scan or in case of stool in the abdominal target drainage.

The primary outcome variable regarding fluid management was the intraoperative positive fluid excess (PFE), measured as the difference out of administered crystalloids and colloids and the urine output. Ascites and blood loss were excluded from the PFE analysis.

### Statistical Analysis

Descriptive statistical variables are expressed as numbers, proportions, and medians as appropriate. Hypothesis testing for differences between two samples was performed using the Mann–Whitney *U* test. Differences were significant at a threshold of ≤ 0.05. All statistical analyses were performed using Minitab Version 18, Minitab LLC., State College, Pennsylvania, USA.

## Results

### General Characteristics

CA included *n *= 185 patients and CB included *n *= 58 patients. A total of *n *= 135 patients (72.97%) in CA and *n *= 40 patients (68.97%) in CB had no residual tumor at the end of debulking surgery. Severe complications (G3–G5) were seen in *n *= 56 patients (30.2%) in CA and in *n *= 10 patients (17.2%) in CB. In total, there were *n *= 117 patients with anastomoses and *n *= 149 anastomoses (*n *= 86 patients with *n *= 1 anastomosis, *n *= 30 patients with *n *= 2 anastomoses, and *n *= 1 patient with *n *= 3 anastomoses). Anastomotic leakage was seen in *n *= 16 patients out of *n *= 91 patients (17.6%) in CA and in *n *= 3 out of *n *= 26 patients (11.5%) in CB (*p *= 0.188). There were *n *= 3 ALs in a total of *n *= 38 anastomoses (7.9%) in CB and *n *= 16 ALs in *n *= 111 anastomoses in CA (14.4%). Only *n *= 1 AL was at a PFE of less than 3000 ml. There was no significant difference between both cohorts regarding surgical complexity, tumor load (by PCI and FIGO stage), ASA score, or the number of intestinal resections and anastomoses. CB patients were significantly older, were less frequently treated with neoadjuvant chemotherapy and exhibited a shorter duration of surgery, as depicted in Table 1. Furthermore, CB patients required significantly less postoperative intensive medical care and less postoperative ventilation. Length of hospital stay was significantly reduced in CB patients (Table 2). CB patients showed no increase in severe (G3–G5) complications (*p *= 0.063) or anastomotic leakage (*p *= 0.556). Ultrasevere complications (G4–G5) were exclusively seen in CA patients (*p *= 0.0255). CA patients received a median of 0 whole blood transfusions (range: 0–14) and a median of 0 fresh frozen plasmas (FFP) (range: 0–18). CB patients received a median of 0 whole blood transfusions (range 0–3) and 0 FFPs (range: 0–8).

**Table 1:** Patient demographics and surgical details of cohorts A and B

Patient characteristics	Cohort A	Cohort B	*p* value
ASA score			0.254
ASA 1/2	127 (68.6%)	35 (60.3%)	
ASA 3/4	58 (31.3%)	23 (39.7%)	
PCI			0.892
Median	9 (range 0–28)	9 (range 3–33)	
FIGO stage			0.931
< IIIC	69 (37.3%)	20 (34.5%)	
≥ IIIC	116 (62.7%)	38 (65.5%)	
SCS			0.551
1–2	110 (59.5%)	37 (63.8%)	
3	75 (40.5%)	21 (36.2%)	
Intestinal resections			0.513
Yes	92 (49.7%)	26 (44.8%)	
No	93 (50.3%)	32 (17.3%)	
Anastomosis performed			0.561
Yes	91(44.8%)	26 (44%)	
No	94 (50.8%)	32 (55.2%)	
Timing of surgery			0.268
Primary	156(84.3%)	52 (89.7%)	
Recurrence	29 (15.7%)	6 (10.3%)	
Median age	61 years (range: 25–86 years)	67 years (range: 38-82 years)	0.038
Duration of surgery			0.001
median	341 min (72–695 min)	249 min (80–571 min)	
Neoadjuvant chemotherapy			0.03
Yes	52 (28.1%)	9 (15.5%)	
No	133 (71.9%)	49 (84.5%)	

Values are shown as median or number of events and percentages

**Table 2:** Postoperative outcome data, intraoperative fluid differences, and noradrenalin amount

	Cohort A	Cohort B	*p* value
Severe complications (G3–G5)	56 (30.3%)	10 (17.2%)	0.063
Ultrasevere complications (G4–G5)	14 (7.6%)	0	0.025
Anastomotic leakages	16 (17.6%)	3 (11.5%)	0.188
First defecation	Day 3	Day 2	0.001
Median length of stay	15 days	12 days	0.003
	(range: 3–197)	(range: 7–68)	
Need of intensive care	1 (range: 0–42)	0 (range: 0–6)	0.001
Need of postoperative ventilation			0.001
Yes	72	1	
No	113	57	
Median PFE	5200 ml	2598 ml	0.001
Median PFE			
PCI ≤ 10	4750 ml	2400 ml	0.001
PCI > 10	6750 ml	3150 ml	0.001
	(*p*: 0.001)	(*p*: 0.006)	
Median PFE			
FIGO I–IIIB	3750 ml	2521 ml	0.001
FIGO IIIC–IVB	6750 ml Difference (*p *= 0.001)	2714 ml Difference (*p *= 0.527).	0.001
Median maximum noradrenalin dosing	6 µg/kg/min;	10 µg/kg/min	0.001

### PFE

Intraoperatively, CB patients received significantly more noradrenalin and had a significantly lower median PFE as depicted in Table 2. Both cohorts showed a significant increase in the PFE in case of a PCI > 10, but again the increase was significantly lower in CB patients. Comparing FIGO stages only, there was no significant increase of the PFE in CB patients. In CA patients there was a significant increase in the case of FIGO stages greater than IIIB.

In general, severe complications (G3–G5) and anastomotic leakage increased with increasing fluid load as depicted in Table 3. Further reasons for significant more severe complications (G3–G5) were a FIGO stage greater than IIIB (*p *= 0.004), a surgical complexity score of > 2 (*p *= 0.027), the presence of anastomoses (*p *< 0.001), and a pre- and postoperative hemoglobin difference of equal to or more than 3 g/dl, as shown in Table 4. AL rates were not affected by increasing hemoglobin differences (≤ 3g/dl; *p *= 1.000, ≤ 4 g/dl; *p *= 1.00, ≤ 5 g/dl; *p *= 0.064). Shorter times to first defecation were seen in CB patients (*p *= 0.001), in case of a PFE less than 5000 ml (*p *= 0.001) and in case of a preoperative bowel preparation with antibiotics (*p *= 0.001). In total there were 37 loop ileostomies (31.6%) performed, 29 in CA and 8 in CB.

**Table 3:** Association of fluid balance with severe complications (G3–G5) and anastomotic leakage

Factor	No. of patients with G3–G5	No. of patients with G0–G2	*p* value
PFE ≤ 3000 ml	16	60	0.034
PFE > 3000 ml	50	123	
PFE ≤ 4000 ml	19	96	0.001
PFE > 4000 ml	47	81	
PFE ≤ 5000 ml	27	114	0.001
PFE > 5000ml	39	63	
	No. of patients with AL	No. of patients without AL	
PFE ≤ 3000 ml	1	23	0.298
PFE > 3000 ml	18	75	
PFE ≤ 4000 ml	1	38	0.006
PFE > 4000 ml	18	60	
PFE ≤ 5000 ml	3	47	0.043
PFE > 5000 ml	16	51

**Table 4:** Association of the postoperative hemoglobin difference with severe complications

Pre- and hemoglobin difference postoperative	No. of patients with G3-G5	No. of patients with G0-G2	*p* value
≤ 3 g/dl	16	72	*p *= 0.024
> 3 g/dl	50	105	
≤ 4 g/dl	30	113	*p *= 0.013
> 4 g/dl	36	64	
≤ 5 g/dl	43	148	*p *= 0.05
> 5 g/dl	23	29	

### Discussion

These two unselected real-world cohorts demonstrate that an intraoperative fluid restriction according to a PPVGHM, achieved by an increased use of noradrenalin, is possible without increasing severe complications in patients with ovarian cancer. In fact, ultrasevere complications (G4/G5) were exclusively seen in CA with liberal fluid management. In general, intraoperative fluid reduction led to earlier postoperative defecation, shorter length of hospital stays, and less intensive care need. No acute kidney injury was observed in the whole PPVGHM cohort.

Fluid overload and fluid restriction are established risk factors regarding postoperative complications in abdominal surgery.^17,18^ Current recommendations of the ERAS society restrict fluids to a maximum postoperative weight gain of 2.5 kg in colorectal surgery.^11^ There is no such recommendation in patients with ovarian cancer yet.^19^ Consistent with the above-mentioned recommendations, we observed a significant increase of complications beginning at a PFE of 3000 ml.^11^ A recent analysis from the Karolinska Institute from Sweden also found increasing complications at a net fluid balance at 3000 ml, 48 h after surgery.^14^ The net fluid balance was defined as the sum of all administered fluids including blood products minus all fluid losses including blood, urine, and ascites.^14^ In our analysis, the positive fluid excess was defined as the sum out of all administered crystalloids and colloids minus the urine output. We did not consider ascites or blood loss to keep the parameter as simple as possible. Ascites was not considered, as this volume is neither intracellular nor intravascular, and therefore excluded from the organ perfusion anyway. Blood loss was not considered as this is, besides a spectrophotometric measurement a rather roughly estimated parameter and therefore of limited reliability especially in patients with advanced-stage ovarian cancer.^20^ Furthermore, advanced-stage ovarian cancer patients show an intraoperative capillary leak and intravascular hypoalbuminemia, consecutively leading to intra-abdominal extravasation and enormous intercompartmental fluid losses.^13^ To maintain hemodynamic stability in these cases, fresh frozen plasmas are recommended to be transfused without resemblance to blood loss.^21^ Consecutive blood replacements are based on intraoperative hemoglobin levels and the amount of fresh frozen plasmas transfused, rather than on the estimation of the blood loss during surgery and the circulatory situation itself. The ESGO guideline recommends a liberal policy regarding the transfusion of blood products.^12^

Recently, two different pathways have been introduced to optimize the intraoperative fluid management in abdominal surgery and ovarian cancer surgery, i.e., goal-directed hemodynamic management and goal-directed fluid management. The goal-directed hemodynamic management is based on pulse pressure variations and stroke volume variations as main indicators of the fluid responsiveness of patients during surgery. In patients with ovarian cancer this led to shorter length of hospitalization, shorter times to first postoperative defecation and lower blood lactate levels, postoperatively.^15^ The goal-directed fluid therapy is based on the near maximal stroke volume as the main indicator for fluid replacement.^22^ In abdominal surgery, for various reasons, no positive effect on the postoperative outcome has been seen so far.^22^ The ESGO guideline of the perioperative management of patients with ovarian cancer defines no cutoff for a maximum of intraoperative fluids so far.^12^ The PPVGHM in CB patients led to a median PFE of no more than 3150 ml even in the high tumor load group with a PCI of ≥ 10. The PPV has been identified as a precise marker for fluid responsiveness, easy to monitor and more reliable than systolic pressure variations or the pulse contour stroke volume variation.^23^ The PPV displays the position on the Frank–Starling curve, where the patient’s heart is operating. If it is operating on the plateau, preload and contractility are at their optimum and the PPV is low. In case of operating on the steep part of the curve, where the preload is decreasing, the PPV increases. Nevertheless there are also several limitations for the use of the PPV regarding volume expansion or volume depletion as cardiac arrythmia, active breathing, and very low tidal volumes.^23^

Regarding ALs, CA showed an AL rate of 17.6% accompanied by a significantly higher median PFE of 5200 ml compared with CB with an AL rate of 11.6% with a median PFE of 2598 ml. In the whole population, significantly more anastomotic leakages were seen at a PFE ≥ 4000 ml. In fact, only one AL was seen at a PFE of less than 3000 ml. The AL rate in CB dropped, despite a more unfavorable population with a median age of 67 and a mean age of 64.5 years and a higher rate of additional small bowel resections of 46.2% by reducing fluids. This compares favorably with Lago et al., who showed that age and additional small bowel resections were significant risk factors for AL in the multivariate analysis.^24^ The AL range at those eight centers of Lago et al. was 1.7–12.5% in a more favorable cohort with a mean age of only 58.7 years and a rate of additional small bowel resections of only 8.9%. CA was also less favorable, with a mean age of 60.5 years and a rate of additional small bowel resections of 20.9%, but also received significantly more fluids. Analyzing the surgical expertise in numbers, our data are comparable with the data of Lago et al.^24^ The average number of patients per center per year with anastomosis was 11.6, considering that 695 patients from 8 centers within 7.5 years were analyzed. In detail, the performance of an anastomosis ranged from 3.5 to 18.7 patients per center per year.^24^ The average number of patients with an anastomosis was 14.6 out of 30.4 patients undergoing cytoreduction per year in our center, in a less favorable cohort.

Regarding anastomoses, the fluid overload leads to at least three consequences. First, the positive fluid excess leads to more intraabdominal pressure and less tissue circulation due to a splanchnic edema..^10,25,26^ Second, the structural and functional stability of anastomoses is altered with increasing fluid quantity, and the needed pressure to accomplish anastomotic bursting decreases.^27^ Therefore, it seems of imminent importance to further explore the entire postoperative fluid management in addition to that, as the first bursting pressure for an anastomosis will be on day 2 or 3 and ALs are generally recognized on days 5 to 8.^28^ The third consequence of a fluid overload by crystalloids is hemodilution, altering anastomotic healing due to a decreased oxygen supply if not balanced postoperatively to hemoglobin levels above 10 g/dl.^29^ In our cohorts we saw an association of pre- and postoperative hemoglobin differences of equal or greater than − 3 g/dl and an increase in severe postoperative complications. We did not observe more ALs, which may be related to the small sample size of ALs and due to a very liberal transfusion of blood within the postoperative course. While preoperative anemia is a known risk factor for postoperative adverse events, postoperative anemia has just recently been identified as risk factor for postoperative complications.^17^ Regarding intestinal anastomoses, it is associated with altered anastomotic healing in the small and the large bowel.^30^ Whether generous whole blood transfusions can modify this observation has not yet been answered due to scarce data regarding this question.

In accordance with previous studies we saw more complications in cases where there was creation of anastomoses, as surgical complexity increased, and as FIGO stage increased.^8,31^

Our study is limited by its retrospective design and its sample size, both potentially being confounding factors. Significant differences between cohorts were the application of neoadjuvant chemotherapy, the age of the patients, and the operating times. The difference in the use of neoadjuvant chemotherapy is due to the prior policy of the clinic to administer neoadjuvant chemotherapy in case of ascites of more than 500 ml until 8/2019. The age difference is most probably due to the retrospective real-world cohorts. No patient scheduled for cytoreduction was excluded. The shorter operating times in cohort B are probably due to the same reason. But of course, we cannot exclude any bias here.

Furthermore, we evaluated only the intraoperative fluid management, excluding the postoperative fluid management until patients were fully recovered. Due to the retrospective design, we were not able to provide a detailed description of the factors leading to fluid management before implementing the new standard of care in 2021.

A strength of our study is the homogeneous patient population and the simple parameters used for optimization of the fluid management, which makes our PPVGHM easy to implement into the routine intraoperative management of ovarian cancer patients. Further prospective studies are urgently needed to optimize the intra- and postoperative fluid management in patients with ovarian cancer. This is emphasized by the fact that fluid management has been recognized as modifiable risk factor, while comorbidities of patients and the goal of complete cytoreductive surgery are set parameters in the fight against postoperative complications and for survival in ovarian cancer patients.
